# Learner pregnancies: views of parents in Madibeng Municipality, North West Province, South Africa

**DOI:** 10.4314/ahs.v22i2.53

**Published:** 2022-06

**Authors:** Tshiamo N Ramalepa, Tendani S Ramukumba, Majapi Elizabeth Masala-Chokwe

**Affiliations:** 1 Nursing Science Department, Sefako Makgatho Health Sciences University, Molotlegi Street Zone1, Ga-Rankuwa, 0208; 2 Adelaide Tambo School of Nursing Science, Tshwane University of Technology Staatsartillerie Road, Pretoria West, Pretoria 0001

**Keywords:** Learner pregnancies, pregnancy in schools, prevention of pregnancy, school stakeholders, views of parents

## Abstract

**Background:**

The learner pregnancy phenomenon is one of a variety of social phenomena faced by schools globally. In South Africa, the phenomenon has been increasing in intensity over the years, as shown in the increasing number of learner pregnancies reported by the Department of Basic Education. Due to its social consequences, learner pregnancy involves different stakeholders, such as teachers, parents and the community.

**Aims:**

The study aimed to explore the views of parents regarding learner pregnancy in schools of Madibeng Municipality, North West Province, South Africa.

**Methods:**

A qualitative, exploratory and descriptive study was conducted using a purposive sampling method. Four focus group interviews were held with a total of 25 participants, and data were analysed using Tesch's approach to content analysis.

**Findings:**

Parents reflected that parents have a responsibility to communicate and share sexual and reproductive information with their children so that the children can make informed decisions about their sexual practices. They further agreed that the responsibility for learner pregnancy management should extend to teachers, parents, nurses, school governing bodies, churches, the community and the government.

**Conclusion:**

Stakeholders such as parents, teachers and the community, as internal or external partners, should play a pivotal role in preventing and managing learner pregnancy in schools.

## Introduction and background

In South Africa, learner pregnancies have been receiving more attention following a report by Jewkes, Morell and Christophides in 2009 that 19.1% of female learners had fallen pregnant at least once before they turned 19 years old^1^. It has been almost 11 years since the 2009 report, and the learner pregnancy numbers are still alarming. The latest report by the Department of Basic Education indicated that 16% of girls between the ages of 16 and 19 had given birth in South Africa and that 15 504 pregnant learners were estimated to be in schools at the time of the report^2^. The number of young girls giving birth in South Africa is distressing because it was estimated that 72 272 girls below the age of 18 years delivered babies in 2015^2^. The pregnancy crisis in schools remains a challenge for teachers, parents and communities in general. However, the Eastern Cape, Limpopo and KwaZulu-Natal provinces have significantly higher learner pregnancy incidences than other South African provinces^3^. Although parental involvement in school learners' education is difficult, parents are still encouraged to be involved in volunteering to assist in the classroom, attend workshops, or attend school plays and sporting events^4^.

The same principle of parental involvement in such school activities should be encouraged in learner pregnancy prevention, management and support. Du Preez et al.^5^ recommend that teachers alone should not be responsible for the health and safety of pregnant learners in the school environment. Therefore, a partnership must be encouraged to develop a policy that encourages parental involvement in learner pregnancy in the school environment. Lack of parental involvement and support during pregnancy can lead to unforeseen negative outcomes such as delayed antenatal booking, pregnancy-related stress, abortion and suicide among pregnant learners^6^. School governing bodies (SGBs) form the bridge between the school management, parents and the community. This enables the SGB to be directly involved in school governance while representing the community. The policy environment has to be enabling and supportive for teachers, SGBs, parents, school health nurses and the community to collaborate in the prevention and management of learner pregnancy.

Parents, as stakeholders, have the responsibility to communicate with their children about the school, participate in school activities, communicate with teachers about their children, and supervise their children at home, which positively affects the children's academic success^4^. However, there is little parental involvement in the prevention and management of learner pregnancy in schools. In a study conducted in Vhembe District, South Africa, Radzilani-Makatu6 found that teenagers without parental involvement and support experienced hardships throughout their pregnancy and childbirth, which in turn affected their health and social lives. These hardships can be perpetuated to academic success and future socio-economic success, which poses a significant threat to degeneration as young women are considered to contribute to the overall social decline through their sexual practices and reproductive status^7^. Parents and teachers form part of the community and represent the community in schools. Nonetheless, the entire community has a specific role to play in prevention and management of learner pregnancy. This study investigated the opinions of parents regarding learner pregnancy in schools of Madibeng Municipality, North West Province.

## Aim and objectives of the study

The aim of the study was to explore the views of parents regarding learner pregnancy in schools of Madibeng Municipality, North West Province, South Africa. The objectives of the study were to explore and describe the views of parents regarding learner pregnancy in schools of Madibeng Municipality, North West Province.

## Methods

This study followed a qualitative, exploratory and descriptive approach. An exploratory study is conducted when little or no previous research has been done about the phenomenon being investigated, and a study is contextual when the study procedures and findings are only relevant to the location where the study is conducted^8^,^9^. The study was conducted in Madibeng Local Municipality, Bojanala District, North West Province, South Africa. Madibeng Local Municipality is located within the Bojanala Platinum District in the North West Province. It is located 50 km north of Pretoria and is one of five municipalities in the Bojanala Platinum District^10^. The 2016 community survey^11^ estimated that the population of Madibeng local municipality was 536 111. Madibeng is a rural municipality which comprises approximately three towns, 43 villages and 9 000 farm areas. There are 146 schools in Madibeng, most of which are in rural areas. Of the 146 schools, 132 are public schools and 14 are private schools^12^. An exploratory and contextual approach allowed the researchers to explore the views of parents regarding the prevention and management of learner pregnancy in schools.

### Population and sampling

The target population was all parents of learners who attended any of the schools located within Madibeng Local Municipality. The inclusion criteria were based on parents of learners who attended any school located within Madibeng, including SGB members, school volunteers and parents in general. Purposive sampling was used to select the sample for the study, as parents were chosen to participate in the study because the primary researcher found them to be knowledgeable about the research topic8. Sampling refers to selecting a sample from the population in order to obtain information about a specific phenomenon in a way that represents the population in which the researcher is interested^13^. Data were collected from a total of four groups of parents, each with between six and eight participants. The sample size was determined by saturation of data, when no new information emerged during data analysis^14^. Saturation of data was achieved with the third group, after which one more focus group interview was conducted to verify that there was no new information emerging. The total number of participants in the four focus groups interviews conducted was 25.

### Data gathering method and process

The data gathering method used in this study was focus group interviews using a focus group interview schedule. Data gathering is a detailed process of gathering of information in order to address the research question, purpose and objectives of the study8. By using the interview method of data collection, the researcher obtained responses from participants in a face-to-face setting, using the focus group interview schedule as a guide^13^. The focus group interviews allowed participants to interact and introduce new concepts to the discussion, the primary researcher acted as the moderator of the discussion. The interview schedule was designed based on the main research question of the study and confirmed by the literature. The focus group interview schedule was pretested with one focus group that was not included in the data analysis. The study was approved by the Tshwane University of Technology Ethics Committee and the North West Department of Education.

The primary researcher went to schools and requested learners to inform their parents about participation in the research study the day before data collection. The following day, groups of parents came to school to participate in the study. Parents who met the criteria for participation in the study were recruited to participate. Parents who worked at the school as supporting staff members, parents who sold food outside the schools and parents who came to school to meet the teachers or principal were included in the study if they met the criteria for participation. The focus group interviews were done on the school premises and in the school boardrooms. Each focus group interview lasted for approximately 30 minutes. An information leaflet and a consent form were provided to each parent before the commencement of the interviews. Those who participated in the focus groups, signed the consent forms. A research assistant and field worker assisted the primary researcher with data collection after signing a confidentiality form. The English and Setswana languages were used to conduct all the interviews. During transcription of the data, Setswana interviews were translated to English by the research assistant. Permission to use a voice recorder was granted by each participant after the primary researcher explained the purpose of using the voice recorder during the focus group interviews. The recorded interviews were transcribed verbatim by the researcher in order to facilitate data analysis.

### Data analysis

Data were analysed by means of qualitative content analysis using open coding according to a data analysis tool designed based on Tesch's approach^15^. Data analysis was done to reduce, organise and give meaning to the data. The primary researcher used the process of content analysis by organising and incorporating narrative, qualitative information according to themes and concepts arising from the collected data^9^,^16^. Parents' responses during the interviews were transcribed verbatim and categorised into themes for analysis. Verbatim transcription was an important step in preparing for data analysis. It was also important to ensure the accuracy of the transcriptions so that they validly reflected the totality of the interviews8. An independent coder, who was a midwife and community health nurse with qualitative research experience, was identified, and all the notes and transcripts were sent to the coder for analysis. Botma et al.^8^ define coding as a process of organising the material into pieces of text before bringing meaning to the information. Data were analysed until saturation was met, when additional data gathered from parents yielded no new information^13^.

### Ethical considerations

The Tshwane University of Technology Ethics Committee approved the research study (Ref: REC/2018/11/005). Because the overarching project included school learners, the North West Department of Education and school principals granted permission for the study to be conducted. All parents provided voluntary informed consent by signing the consent form. Furthermore, privacy and confidentiality were ensured by not publishing the participants' names in any official report, as well as by conducting the interviews in a private room. Measures to ensure trustworthiness were followed. These included prolonged engagement, which was achieved through the primary researcher's familiarity with the research context and participants as he had worked at one of the local clinics for three years. Furthermore, the primary researcher introduced himself to the participants, explained all the procedures, answered all questions and built a rapport before data collection. The project was peer reviewed throughout the study. The study was guided by qualified supervisors who were knowledgeable and had experience in research methodology and community nursing. The Ethics Committee of the Tshwane University of Technology and approved the research project. The interview schedules were pretested before commencing with data collection. The criteria for participation were clearly explained, with inclusion and exclusion criteria being described to enable effective auditing of the data collected throughout the study. The researcher displayed fairness when recruiting participants by being transparent and giving potential participants an equal chance to participate. Furthermore, the interview transcripts, audiotapes, voice recordings and field notes will be kept for five years as an audit trail.

## Results

The demographic data for parents included the number of SGB members, school volunteers and parents who are not active members in schools. There were four focus groups, with a total of 25 participants. The groups consisted of one SGB member, two school volunteers and 22 parents. The focus groups included nine male and 16 female parents. Four themes are discussed in this paper, namely parental responsibilities to the child and grandchild, parental disillusionment, barriers to supporting learners and stakeholder involvement.

### Parental responsibilities to the child and grandchild

The results from parents suggested that all parents have the responsibility to communicate and share information with their children. They agreed that this would assist learners to make informed decisions regarding their sexual practices. Parental communication and guidance were some of the factors suggested to prevent learner pregnancy. Participants stated that, if parents started to communicate with their children, children would be more informed and would be able to make good decisions regarding sexual practices. The following are some of the comments made by parents:

“*It is to talk with them [learners] about everything, it's to talk with them about pregnancy, so we should share everything with our children so that they are aware that when they have sex, they will get pregnant*.” FG2P1

“*The bottom line is that it all starts from parenthood, if there is poor communication and engagement with the child, the child starts doing things that are out of order and eventually becomes pregnant without having the experience and knowledge about the consequences of pregnancy. Some of the parents are not open to socialising their children with these issues and this affects the society as whole*.” FG1P1

Parents indicated that, when a learner became pregnant at a young age, the responsibility for the baby fell on the learner's parents, which was a burden to them. One parent reflected that it was painful to buy nappies with one's first salary while one's friends were driving nice cars. Other parents felt that it was difficult to take care of their own children and grandchildren at the same time. Two parents stated the following:

“*This issue is not nice because when they get pregnant we take responsibility for it, the child becomes yours. It is painful to have to buy nappies with you first salary while your friends drive nice cars enjoying their lives.*” FG3P5

“*Sometimes without even looking at my situation whether I have something sustainable to provide for the child that she is bringing into this earth or will it be the responsibility of the [mother's] parents. Sometimes a child in the family has a baby, she is still supported by her parents and goes to school, so who is going to take care of the grandchild*.” FG1P1

### Parental disillusionment

Parents indicated that learner pregnancy affected all family members. They felt that it was embarrassing and disappointing to have a child pregnant while she was still in school, although they were often optimistic about the future of the girl-child. Furthermore, they indicated that experiencing learner pregnancy was demotivating because they had high hopes for their children and wanted their children to succeed in life. Parents suggested that encountering learner pregnancy in the family led to disillusionment and embarrassment. The disillusionment stemmed from the premise that parents were always hopeful that their children would succeed in life, and learner pregnancy was seen as a negative occurrence within society. According to parents, learners who became pregnant ended up disappointing their families. Some of the parents' comments are provided below:

“*It affects the family in a sense that she disappoints the family as they had high expectations from her, so when they realise that she is pregnant and is bringing a child to this world, and at the same time they were hopeful about her education, so it is very disappointing*.” FG3P3

“*It delays the child to complete school, you find that she is left behind because she has to take care of the child, and as a parent you become embarrassed and you find it difficult to talk to people because you are embarrassed, I can say that it affects the whole family as the whole family becomes embarrassed, we have high expectations for our children. Some don't go back because they feel embarrassed and think about what are the teachers going to say and what are the learners going to say*.” FG2P1

Parents showed positive persistence. Despite being disappointed and embarrassed by learners who became pregnant, they indicated that they wished their children all of the best in their futures. Additionally, although parents were concerned about the impact of learner pregnancy on their children's futures, they remained hopeful about their children overcoming pregnancy-related adversities by going back to school to complete their education. Some parents indicated that, by reprimanding their children about pregnancy while in school, they were looking out for the children's future, while other parents agreed that pregnant learners should be able to complete schooling. Some of the comments by parents are provided below:

“...*our children should have better lives, they should be able to finish school and go to universities because bursaries are available now, and these things of learner pregnancy needs to stop because if it does not stop then many of them will fall into the trap*.” FG1P5 “*With some you find that you had some money saved for them to complete school but then they can get pregnant, which affects their futures...the issue is that what happens to their future. You as the parent give everything to your child in order for them to be better than you*.” FG3P1

### Barriers to supporting learners

Parents suggested that learners had to be informed about reproductive health issues and pregnancy so that they could be wiser with their sexual choices. Lack of information and difficulty in communication between teachers, parents and learners were identified as some of the problems leading to learner pregnancy. Although providing information and teaching learners about sex and reproductive health were identified as measures which could lead to pregnancy prevention, parents identified barriers which made it difficult for these measures to be effective. It was agreed that it was difficult to talk to learners because learners thought they already knew everything. Sharing information and teaching should not only come from teachers, because parents, nurses and the community as a whole can play a major role in providing this kind of support to learners. Some parents indicated that teaching learners, especially girls, was critical so that they would be careful when they were involved in sexual practices. The following comments were made:

“*It is all about teaching our children especially the girls that when they start seeing their periods they should not sleep with boys. We should inform them that prevention alone is not enough because there are diseases like HIV out there. So we should teach our children from a young age and should not be embarrassed to talk about these issues with them*.” FG2P1

“*It's up to us, the community and the professionals as well to come in the open and talk and come up with solutions, analyse our children and discuss such topics. Then afterwards we should share the information with the learners, I think then this thing of teenage pregnancy will be limited, it would not end but it will be limited and controllable. When we don't speak it's like we are creating another problem*.” FG1P1

Even though communicating and sharing information with learners could assist them in making informed decisions regarding their sexual practices, parents identified factors which formed barriers to supporting learners. Such factors included that learners thought they already knew all the information their reproductive health and that some learners did not want to use contraceptives. For example, the following comments were made:

“*My child who was born in 2004 does not want to ‘prevent’ because she says she does not date, she does not even want any kind of injection, so when she starts dating it's going to be a problem because she will get pregnant and get diseases because she does not want to prevent*.” FG2P3

“*Another thing is that all the learners receive information, I am telling you now that there is no learner who doesn't receive information about sexual orientation, but according to the way they act; it is beyond their knowledge*.” FG1P6

According to parents, it was difficult to support learners through communication, teaching and providing information because some learners thought they knew better, some were ignorant and some were not well informed. It is important to provide learners with other means of support to supplement teaching, such as the involvement of other relevant stakeholders.

### Involvement of stakeholders

According to parents, relevant stakeholders such as nurses, teachers, SGBs and the community could play a key role in the prevention and management of learner pregnancy in schools. They indicated that, if such stakeholders took ownership of learner pregnancy and collaborated, it might help in reducing the problem in schools.

Parents further suggested that teachers were trying, but needed help from nurses, SGBs and the community in order to deal with the problem. Some of the comments are provided below:

“*I think there should be nurses in schools who at times should take girl learners and teach them about sexual intercourse and pregnancy, they should also teach them that prevention is not permission to start dating boys, they should focus on their studies and use protection when having sex, at the right and acceptable age. These nurses can check the girls, you know sometimes a girl can be raped and keeps quite about it, so when they are with a nurse they can disclose such information*.” FG2P2

“*Nurses can bring injections to school and inject everyone, they should also start from the age of 9 years and teach our children about sex and what to expect, so that they can all be aware of everything before engaging in such activities*.” FG4P5

The role nurses could play in the prevention and management of learner pregnancy in schools was recognised by parents as they reflected that nurses could assist teachers in educating learners and providing pregnancy prevention measures to all learners. Parents suggested that, even though teachers were trying their best to provide support to learners, they still needed assistance from other stakeholders. Another suggestion was that teachers and schools needed to do more to occupy learners' time with schoolwork. Parents said the following:

“*I think that in schools each class should have a file, each teacher must have a file containing all female learners. On each specific date the teacher should record and keep the dates for prevention, then remind learners and their parents about the prevention date. We must work together, form the school, to the parents and the school and this will make things right*.” FG4P3

“...*the teachers in school must take part and encourage learners on how to solve these issue. The parents should not be the only ones taking responsibility, even the teachers should play a part by teaching them, especially the boys because they should be taught about the effects of sleeping with a girl and that it ruins the girl's future once she gets pregnant*.” FG4P1

Participants indicated that teachers and parents needed assistance, because other stakeholders such as SGBs and the community had to take part in learner pregnancy prevention as well. SGBs form an integral part of both school management and the community, which means that SGBs have influence with regard to decision-making and initiatives in schools. Parents suggested that schools should “choose a group of parents and SGB to be responsible for learner pregnancies in school”. They further indicated that the government could also help prevent and manage learner pregnancy. SGBs, the community, parents, teachers and nurses were some of the stakeholders mentioned by parents, although one SGB member indicated that the SGB did not participate in this issue. Parents said the following:

“*Maybe they should choose a group from parents or SGB who will be responsible for it...in that group there should be a nurse who will assist them. We should be close with the teachers in schools, the connection between teachers and parents can help with everything, and we should not put everything on the teachers' shoulders because they are in school with our children. We shouldn't just sit there and relax*.” FG3P3

“*How are we engaging our children as the community, as parents, as teachers and as the society because it is now becoming our responsibility and the government's responsibility? It's up to us, the community and the professionals as well to come in the open and talk and come up with solutions, analyse our children and discuss such topics. The government should make it a point that learners should not fall pregnant in school, because they will be breaking the code of conduct when it comes to learnership*.” FG1P1

One SGB member suggested that the SGB was inactive in addressing learner pregnancy in schools because the Department of Health was not involved with the SGB. The participant said:

“*At the moment we don't play a major role because we never really receive any information, there should be someone who gives us information and guides us about how to handle such issues when we encounter them. The Department of Health are not involved at all with us*.” FG1P5

The role of stakeholder collaboration was identified as an important measure to prevent and manage learner pregnancy in schools. Parents suggested that educating learners and dealing with learner pregnancy should not be the sole responsibility of teachers, parents or nurses. Instead, SGBs, churches, communities and the government should collaborate with teachers, parents and nurses to address the problem.

## Discussion

The aim of the study was to explore the views of parents regarding learner pregnancy in schools of Madibeng Municipality, North West Province, South Africa. The findings indicated that parents viewed learner pregnancy as a problem for the whole family, although they agreed that the responsibility should be shared by different stakeholders such as teachers, nurses and the community. In a systematic review study, Oyodele, Wright and Maja^17^ found that effective learner pregnancy programmes include the engagement and education of parents and communities in creating opportunities for youth empowerment and youth development initiatives that are contextualised to meet the needs of each community. Each community has unique characteristics that contribute to the burden of learner pregnancy. These characteristics commonly stem from culture, religion and socio-economic background. This study found that lack of information, lack of communication between parents and their children, as well as the negative attitudes of learners, contributed to learner pregnancy. Similar characteristics which predispose communities to learner pregnancy were identified by Masilo and Makhubele, including lack of information, lack of sexual communication among parents and children, poverty, and other family-related circumstances. In addition, one parent in the current study indicated that their child was pregnant because of rape, and yet the pregnant learner experienced the same type of reaction as other pregnant learners from other learners, teachers and the community^7^. The study results showed parental concerns and the difficulties parents experienced when dealing with the aftermath of gender-based violence, such as rape.

Although these factors are common throughout societies, each community has its own unique problems which influence learner pregnancy. Thus, stakeholders who are engaged in programmes related to the prevention and management of learner pregnancy should be community members and community leaders who are familiar with the community's specific problems. This study suggested that nurses need to visit schools regularly to address reproductive health issues and learner pregnancy, and that teachers are not the relevant people to address learner pregnancy in schools. It was stated that “if nurses [could] go to schools for immunisation”, then they could do the same by going to schools for the provision of family planning services and health education. According to Bahraminejad et al.^19^, community-based health promotion programmes require a collective effort from all stakeholders, including community members and various professionals, such as nurses and social workers, to ensure that all local agendas and problems are addressed. The Integrated School Health Policy requires that community members such as parents, nurses, teachers and learners be involved in school health promotion programmes to prevent and manage learner pregnancy.

The management level of each organisation is judged on its ability to recognise that each individual's interests, abilities and resources can contribute to the success of the whole^20^. Consequently, there is a growing cognisance of the importance of recognising and taking into account the perspectives and experiences of people other than researchers, academics and policymakers when determining the relevance and importance of programmes and interventions^21^.

Stakeholders, as internal or external partners, should be allowed to play a pivotal role in schools. Parents should lead the way for stakeholder involvement because they serve as both internal and external stakeholders. Part of the school management is the SGB, which mostly consists of parents and other community members. The stakeholder involvement process provides a locally acquired information base for developing programmes and interventions which are aligned with the views of the community that is being addressed, providing a comprehensive and effective approach to applying such interventions^21^. This gives an opportunity for community members to discuss, plan and implement solutions which they have agreed on, which provides a sense of accountability and responsibility. This approach to health promotion and community engagement can be applied to the prevention and management of learner pregnancy in schools.

## Recommendations

The following recommendations are given based on the study's findings:

- Learner pregnancy prevention and management should be addressed by introducing services which encourage partnerships amongst relevant stakeholders such as parents, teachers, learners, nurses and the community as whole.
- Nurses should be encouraged to visit schools to provide reproductive health education and services, conversely, clinics have to provide youth friendly services to learners who visit the clinic for reproductive health services.
- Teachers, parents and nurses should form a working relationship and introduce a learner reproductive health referral and follow-up system in order to monitor learners' reproductive health needs at home, schools and the clinic.

## Conclusion

This study was justified by its aim, which was to determine the views of parents regarding learner pregnancy in schools. The findings of this study revealed that there are still gaps within the effectiveness of sexual and reproductive health in schools and the community. These gaps can be attributed to the difficulty of conveying the message of reproductive health education to learners because of the barriers identified by parents in this study. Lack of information, communication and parental guidance, as well as ignorance regarding sexual and reproductive health, were some of the barriers identified. Learner pregnancy is not only a problem in schools, because this study revealed that it is a social phenomenon which affects the family and the community as well. Community health care nurses have access to and knowledge of learner pregnancy prevention and management measures, and have a key role to play in the provision of reproductive health services in schools. Moreover, stakeholders, as internal or external partners, should be allowed to play a pivotal role in the prevention and management of learner pregnancy in schools. Parents, as custodians of the community, have to collaborate with teachers and community health nurses in leading sexual and reproductive education and other preventative initiatives to combat learner pregnancy. Conversely, community health nurses, as the custodians of health in the community, should work closely with parents, teachers and the community to address the prevention and management of learner pregnancy in schools.
